# Long-term remission of microsatellite instability-high adenosquamous carcinoma in gastric antrum: a case report

**DOI:** 10.3389/fonc.2025.1516966

**Published:** 2025-04-11

**Authors:** Peng Zhang, Jing Yang, Yongyong Liu, Qing Zhou, Caiqiang Xue, Bin Zhang, Yumin Li

**Affiliations:** ^1^ Department of Pathology, Lanzhou University Second Hospital, Lanzhou, Gansu, China; ^2^ Department of Pathology, Chengdu Integrated Traditional Chinese Medicine (TCM) and Western Medicine Hospital, Chengdu, Sichuan, China; ^3^ Department of General Surgery, Lanzhou University Second Hospital, Lanzhou, Gansu, China; ^4^ Department of Radiology, Lanzhou University Second Hospital, Lanzhou, Gansu, China; ^5^ Gansu Province Key Laboratory of Environmental Oncology, Lanzhou University Second Hospital, Lanzhou, Gansu, China

**Keywords:** long-term remission, microsatellite instability-high, adenosquamous carcinoma, gastric antrum, case report

## Abstract

**Background:**

Gastric adenosquamous carcinoma (ASC) is an exceedingly rare neoplasm. Its infrequent occurrence has resulted in sparse literature on its diagnosis and management, presenting considerable challenges for clinical practice.

**Case presentation:**

A 70-year-old male presented with epigastric pain and, upon gastroscopy and CT imaging, was found to have a mass in the gastric antrum. Histopathological analysis confirmed the diagnosis of adenosquamous carcinoma. Immunohistochemical staining revealed a loss of PMS2 and MLH-1 expression, while molecular analysis confirmed MLH-1 methylation, suggesting a microsatellite instability-high (MSI-H) phenotype. The PD-L1 combined positive score (CPS) was remarkably elevated at 80. Postoperatively, the patient received six cycles of oxaliplatin in conjunction with PD-1 inhibitor therapy. At the one-year follow-up, the patient remained in long-term remission, with no evidence of recurrence.

**Conclusion:**

This case underscores the potential efficacy of integrating surgery, chemotherapy, and immunotherapy in managing gastric ASC, particularly in the context of MSI-H and elevated PD-L1 expression. It further emphasizes the critical role of comprehensive molecular profiling in guiding personalized therapeutic strategies for such rare malignancies. Further research and additional case reports are imperative to establish optimal management protocols for gastric ASC and to enhance long-term outcomes.

## Introduction

Gastric cancer is among the most prevalent malignancies globally, with disproportionately high incidence and mortality rates in East Asia (1). With the development of technology, the internet of things (IOT) has brought significant efficacy and new opportunities for the surgical treatment of gastric cancer, which is the future trend of radical gastric cancer surgery (2, 3). Histologically, gastric cancer encompasses various subtypes, with adenocarcinoma being the predominant form, whereas squamous cell carcinoma remains relatively uncommon. Adenosquamous carcinoma (ASC), a neoplasm comprising both adenocarcinoma and squamous cell carcinoma components, is exceptionally rare, constituting less than 0.5% of all gastric cancers (4). This tumor exhibits complex biological behavior and is typically associated with a poor prognosis (5).

Owing to its rarity, the literature on the diagnosis and treatment of gastric adenosquamous carcinoma remains sparse. Clinical management frequently depends on treatment paradigms derived from conventional gastric cancer cases (4). In recent years, notable advancements in gastric cancer treatment have emerged, particularly with the advent of immunotherapy, including PD-1/PD-L1 inhibitors, alongside traditional surgical and chemotherapeutic approaches (6). Several potential biomarkers, such as Combined Positive Score (CPS), deficient mismatch repair (dMMR), and Epstein-Barr virus-encoded small RNA (EBER), have been identified to guide immunotherapy applications (7). Emerging studies indicate that gastric adenosquamous carcinoma may present with elevated CPS scores and dMMR, providing new therapeutic avenues for this rare malignancy (8).

This case report presents the diagnostic and therapeutic course of a 70-year-old male diagnosed with gastric adenosquamous carcinoma. Postoperative histopathology confirmed a moderately differentiated neoplasm comprising both squamous cell carcinoma and adenocarcinoma components, accompanied by immunohistochemical loss of PMS2 and MLH-1 expression, alongside MLH-1 gene methylation. The patient subsequently received adjuvant chemotherapy combined with immunotherapy (oxaliplatin and sintilimab), ultimately achieving long-term remission. Through a detailed analysis of this case, we aim to elucidate the diagnostic challenges, the influence of molecular characteristics on treatment.

## Case presentation

**Figure 1:**
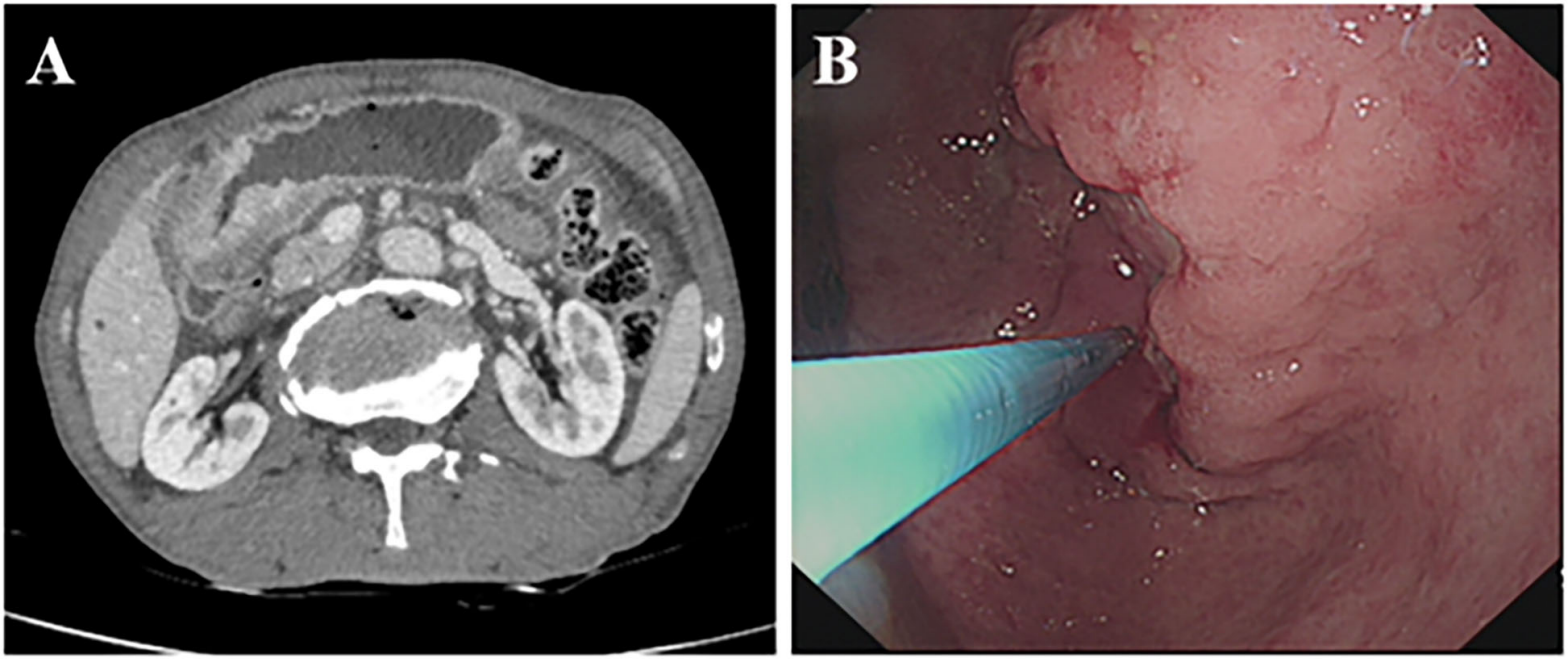
Preoperative imaging and gastroscopy examination. **(A)** Enhanced CT showing thickening of the gastric wall on the side of the antrum lesser curvature, unclear plasma membrane surface, and obvious enhancement of the mass in the portal stage. **(B)** Gastroscopy showing irregular ulcer-type lesion in the antrum.

A 70-year-old male presented with mild epigastric pain, first noted two months prior to admission. CT imaging revealed thickening of the gastric wall in the antrum, with marked contrast enhancement (**Figure 1A**). Gastroscopy demonstrated an irregular, ulcerative lesion in the gastric antrum, characterized by poorly defined borders and surface bleeding (**Figure 1B**). Biopsy confirmed squamous cell carcinoma, while serum tumor markers (CEA, CA199, CA125, CA724, AFP) were within normal ranges. The patient subsequently underwent laparoscopic distal gastrectomy with lymphadenectomy. Intraoperatively, a 7 cm tumor was identified on the lesser curvature of the gastric antrum, extending through the serosa and adherent to the transverse mesocolon.

**Figure 2:**
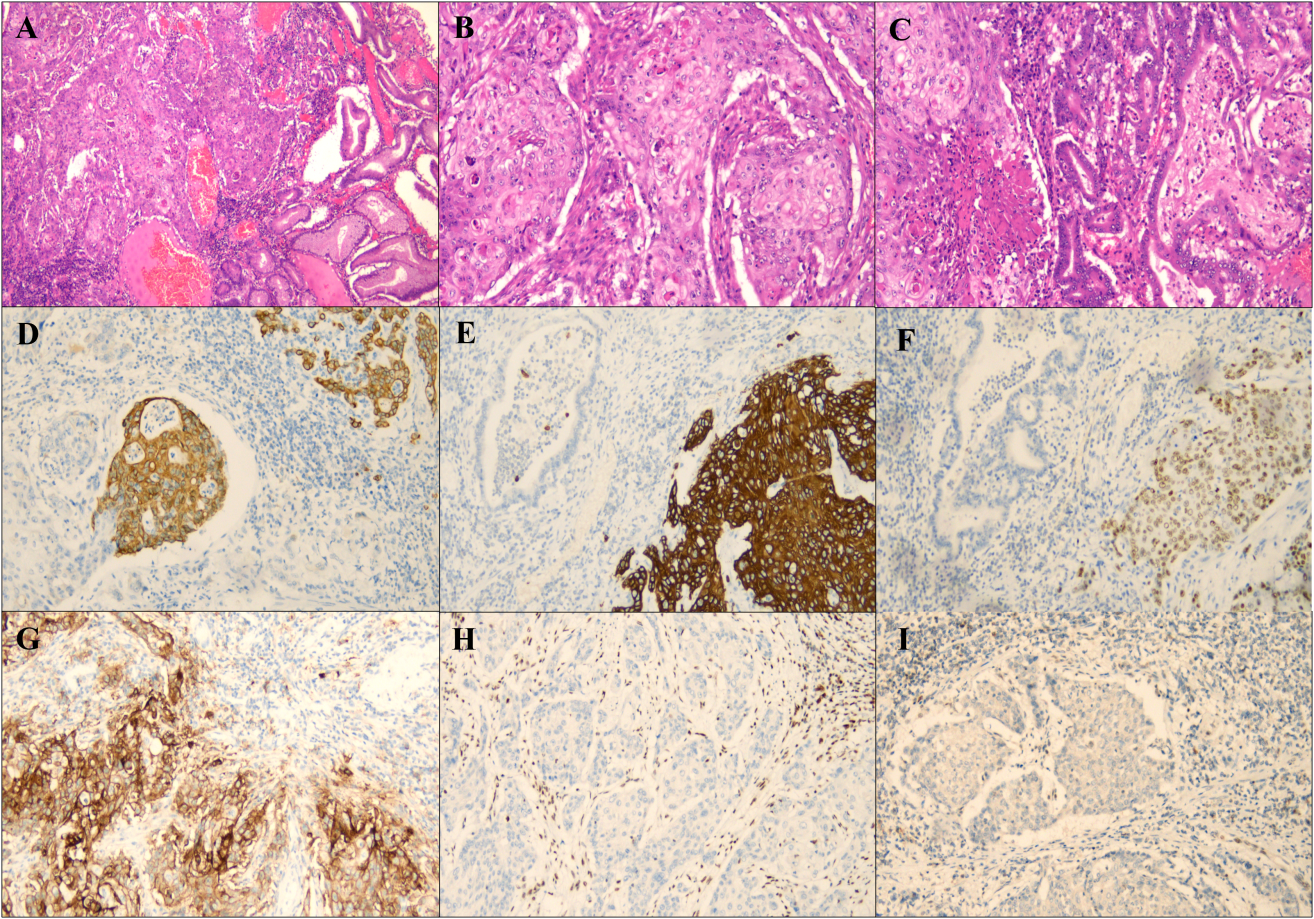
Histological and immunohistochemical staining of the tumor. **(A)** Both squamous cell carcinoma and adenocarcinoma components were seen at low magnification (magnification×40). **(B)** Squamous cell carcinoma area (magnification×100). **(C)** Adenocarcinoma area in the center. (magnification×100). **(D)** Tumor cells with MLH-1 staining negative for immunohistochemistry (magnification×100). **(E)** Tumor cells with PMS2 staining negative for immunohistochemistry (magnification×100). **(F)** PDL1 immunohistochemical staining (magnification ×100). **(G)** Tumor cells with CK8/18 staining negative for immunohistochemistry (magnification×100). **(H)** Tumor cells with CK5/6 staining negative for immunohistochemistry (magnification×100). **(I)** Tumor cells with P63 staining negative for immunohistochemistry (magnification×100).

Postoperative histopathology revealed a tumor composed of 80% squamous cell carcinoma and 20% adenocarcinoma, both of which were moderately differentiated (**Figures 2A–C**). The tumor infiltrated the full thickness of the gastric wall and involved adjacent vasculature and nerves, with negative surgical margins and metastasis to 1 of 23 lymph nodes. Immunohistochemical analysis demonstrated CK8/18 (**Figure 2D**) and CKp expression in adenocarcinoma, CK5/6(**Figure 2E**) and p40 (**Figure 2F**) expression in the squamous cell carcinoma, and a PD-L1 (**Figure 2G**) CPS score of 80. The tumor exhibited loss of MLH-1 (**Figure 2H**) and PMS2 (**Figure 2I**) expression, while HER2 and EBER testing returned negative results (**Supplementary Materials 1**). P53 showed mutant phenotype (**Supplementary Materials 1**). Further molecular analysis confirmed MLH-1 methylation (**Supplementary Materials 2**), indicating that the MSI-H status was attributable to this methylation (**Supplementary Materials 3**). Therefore, Lynch syndrome was excluded. Postoperatively, the patient received 6 cycles of chemotherapy with oxaliplatin and sintilimab and remained disease-free at the one-year follow-up.

## Discussion

Gastric adenosquamous carcinoma is an exceedingly rare malignancy, most frequently localized in the distal third of the stomach. It is characterized by the presence of both adenocarcinoma and squamous cell carcinoma components, with the squamous component comprising more than 25%. Diagnosis is dependent on histopathological evaluation and immunohistochemical staining to distinguish between the two cellular components (9). The adenocarcinoma component typically displays glandular architecture, while the squamous cell carcinoma exhibits classical features such as keratinization and intercellular bridges. Immunohistochemical analysis demonstrates CK8/18 and CKp expression in adenocarcinoma, and CK5/6 and p40 expression in the squamous cell carcinoma component (10). In this case, both the histological morphology and immunophenotypic profile align with typical adenocarcinoma and squamous cell carcinoma features, providing robust evidence for the pathological diagnosis. By searching Pubmed, we found 2 articles on adenosquamous carcinoma of the digestive system with microsatellite instability and found cases described in English (**Table 1**) (11, 12). And Liu et al. demonstrated Microsatellite instability-high pancreas adenosquamous carcinoma with postoperative liver metastasis recurrence treated with multimodality therapy achieving complete pathological response (12).

**Table 1:** Adenosquamous carcinoma of the digestive system with microsatellite instability.

Reference	Country	Age	Gender	Tumor Location	Primary Symptom(s)	Molecular Features	Treatment Modalities	Follow-Up (months)	Outcome
Rafael Parra-Medina et al. (11)	Colombia	43	Male	Ascending colon	Abdominal pain, hematochezia	MSI-H (MLH1/PMS2 loss)	Right hemicolectomy	NA	No survival data reported
Qinghua Liu et al. (12)	China	48	Male	Pancreatic head	Jaundice, weight loss	MSI-H (MLH1/PMS2 loss)	TACE+Gemcitabine/Albumin-paclitaxel+Sintilimab+Bevacizumab	24	Complete remission
Current	China	70	Male	Gastric antrum	Epigastric pain	MSI-H (MLH1/PMS2 loss+MLH1 Methylation)	Laparoscopic distal gastrectomy+Oxaliplatin (6 cycles) +Sintilimab	12	Disease-free survival

Owing to its rarity, there is a lack of systematic research on gastric adenosquamous carcinoma, and its pathogenesis remains poorly understood. Several hypotheses have been suggested: (1) malignant transformation of ectopic squamous epithelium in the gastric mucosa (13); (2) abnormal differentiation of gastric mucosal stem cells, which typically differentiate into function-specific tissues, resulting in concurrent transformation into both squamous cell carcinoma and adenocarcinoma (14); (3) chronic gastritis, Helicobacter pylori infection, or prolonged chemical exposure (e.g., alcohol) may lead to abnormal epithelial regeneration and squamous metaplasia, eventually culminating in adenosquamous carcinoma (15). In summary, gastric adenosquamous carcinoma likely results from a multifactorial etiology, and further research is essential to elucidate its pathogenesis and provide more robust support for clinical diagnosis and therapeutic approaches.

Given its rarity, treatment strategies for gastric adenosquamous carcinoma frequently draw upon therapeutic approaches utilized for gastric adenocarcinoma and squamous cell carcinoma. Studies reviewing the clinical characteristics of gastric adenosquamous carcinoma suggest that radical surgery remains the preferred treatment for patients with early-stage or locally advanced disease. However, given the high rates of postoperative recurrence, adjuvant therapy is generally required. Postoperative adjuvant chemotherapy is typically modeled on gastric adenocarcinoma protocols, such as oxaliplatin combined with fluoropyrimidine-based regimens like FOLFOX or XELOX (16). Platinum-based chemotherapy regimens have been shown to significantly prolong both disease-free survival (DFS) and overall survival (OS) in patients with advanced-stage gastric adenosquamous carcinoma (5).

Immunotherapy, particularly PD-1/PD-L1 inhibitors, has garnered significant attention in treating MSI-H or dMMR tumors. Microsatellite instability (MSI) refers to alterations in repetitive microsatellite sequences arising from defects in DNA mismatch repair mechanisms, commonly associated with mutations or methylation of genes such as MLH1, MSH2, MSH6, and PMS2. MSI is prevalent in numerous cancers, particularly colorectal and gastric adenocarcinomas (17). Notably, studies have yet to report an association between adenosquamous carcinoma and MSI-H. In several cancers, MSI-H or dMMR status is closely associated with elevated PD-L1 expression, and MSI-H/dMMR tumors often exhibit favorable responses to immune checkpoint inhibitors. In this case, the patient’s MSI-H status and elevated PD-L1 expression likely contributed to the favorable response to sintilimab (18).

## Conclusion

This case report underscores the diagnostic and therapeutic challenges associated with gastric adenosquamous carcinoma, a rare and aggressive subtype of gastric cancer. Notably, the introduction of immunotherapy, in the setting of mismatch repair deficiency, facilitated a sustained remission. Future case reports, combined with advances in molecular pathology and personalized therapeutic strategies, are expected to further enhance the prognosis of gastric adenosquamous carcinoma.

## Data Availability

The original contributions presented in the study are included in the article/**Supplementary Material**. Further inquiries can be directed to the corresponding author.
