# Supplementary figures and images for: Long-term remission of microsatellite instability-high adenosquamous carcinoma in gastric antrum: a case report
Source: Front Oncol. 2025 Apr 11;15:1516966. doi: 10.3389/fonc.2025.1516966 (PMC12021638; doi:10.3389/fonc.2025.1516966)

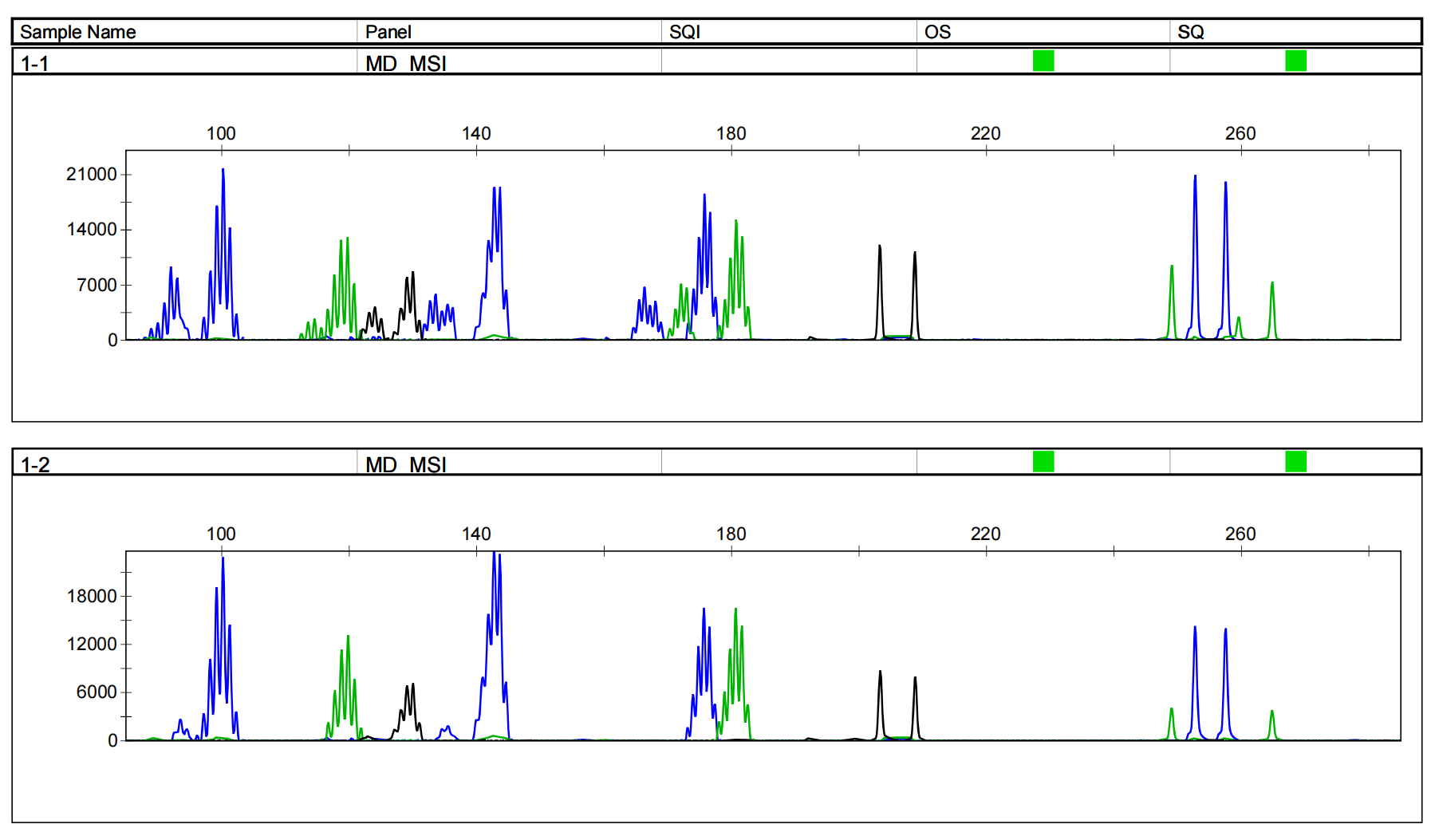

Supplement: Supplementary file 2 [file Image1.tif]

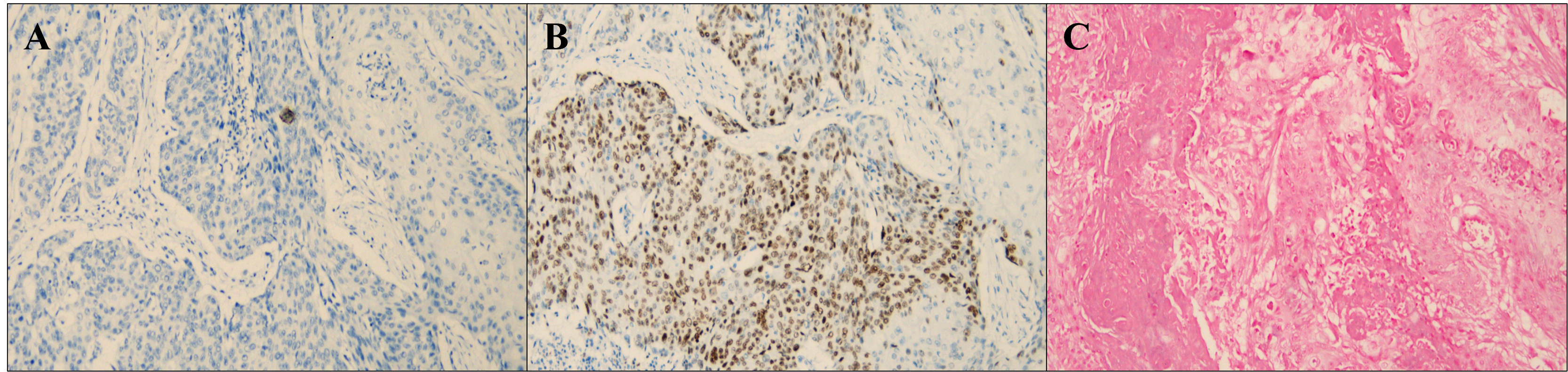

Supplement: Supplementary file 3 [file Image2.tif]

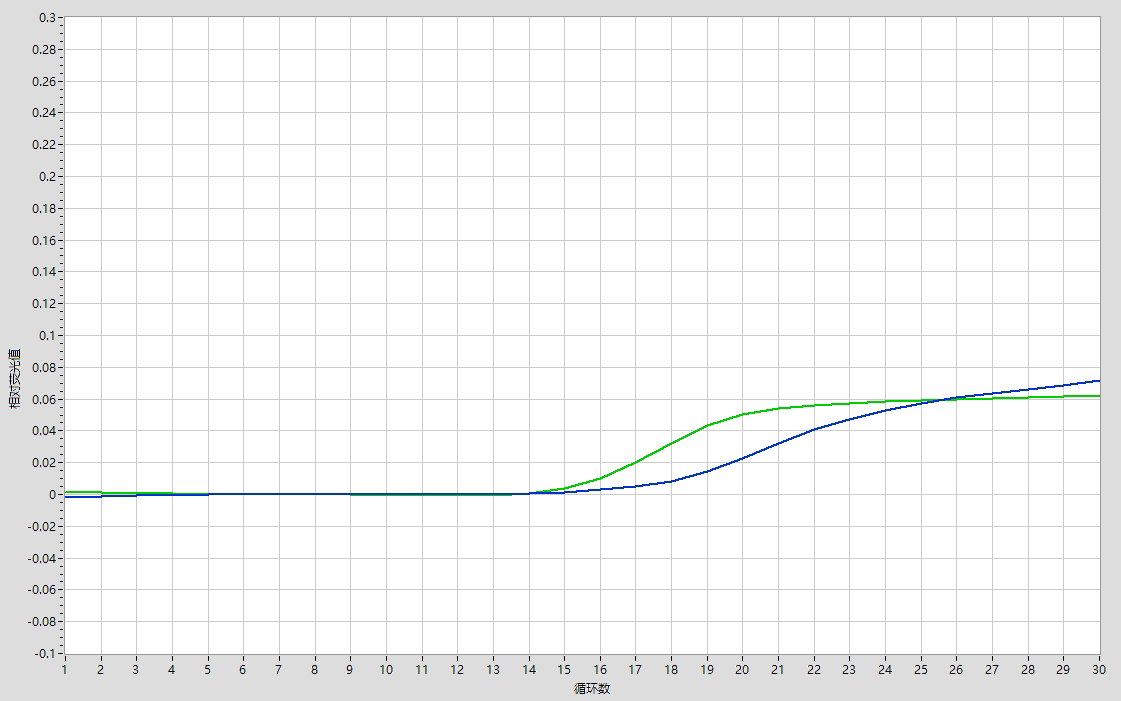

Supplement: Supplementary file 4 [file Image3.tif]
